# Benefit of Salbutamol for the Treatment of Neuromuscular Junction Dysfunction in Patients With Purine-Rich Element Binding Protein A (PURA) Syndrome

**DOI:** 10.7759/cureus.112808

**Published:** 2026-07-16

**Authors:** Maggie L Yau, Cara Beck, Eva L Fung, Eppie M Yiu, Chiara Tewierik, Chun-Him Leung, Hon-Ming Cheung, Karen K Yam, Ka-Nam Wong, Thomas S O'Neill, Katherine B Howell, Shuk-Ching Chong

**Affiliations:** 1 Paediatrics, The Chinese University of Hong Kong, Sha Tin, HKG; 2 Clinical Genetics Service Unit, Prince of Wales Hospital, Sha Tin, HKG; 3 Victorian Clinical Genetics Services, Murdoch Children's Research Institute, Melbourne, AUS; 4 Neurology, The Royal Children's Hospital, Melbourne, AUS; 5 Neurosciences Research, Murdoch Children's Research Institute, Melbourne, AUS; 6 Paediatrics, The University of Melbourne, Melbourne, AUS; 7 Physiotherapy, The Royal Children's Hospital, Melbourne, AUS; 8 Physiotherapy, Prince of Wales Hospital, Sha Tin, HKG; 9 Centre of Medical Genetics, Joint Baylor-CUHK, The Chinese University of Hong Kong, Shatin, HKG

**Keywords:** congenital myasthenia syndrome, genetic syndromes, neuromuscular diseases, pura syndrome, treatment choices

## Abstract

Purine-rich element-binding protein A (PURA) syndrome is a rare neurodevelopmental disorder caused by pathogenic variants in the PURA gene and is characterized by neonatal hypotonia, feeding difficulty, respiratory dysregulation, and severe developmental impairment. The PURA gene has recently been identified as a cause of congenital myasthenic syndrome.

We report two unrelated infants with genetically confirmed PURA syndrome who were treated with neuromuscular junction-directed therapy and reviewed three additional published cases. All five patients had neonatal hypotonia and apnea or respiratory failure requiring respiratory support. Pyridostigmine was used in four of the five infants included with heterogeneous response, while salbutamol, used in three patients, was associated with clinical benefit in all.

These findings suggest that neuromuscular junction dysfunction may contribute to the respiratory and motor phenotype in patients with PURA syndrome. Early consideration of salbutamol, with careful monitoring and objective outcome measurement, may help stabilize severe infantile respiratory failure and improve motor function.

## Introduction

Purine-rich element-binding protein A (PURA) syndrome is caused by heterozygous pathogenic variants in the PURA gene, which encodes the PUR-alpha (PURA) protein that has a critical role in transcription initiation and RNA translation [1]. PURA syndrome is characterized by neonatal-onset hypotonia, feeding difficulty, respiratory problems, hypersomnolence, epilepsy, and severe neurodevelopmental impairment [2]. In a meta-analysis by Reijnders et al. [2], neonatal respiratory problems were reported in up to 57% of affected individuals, reinforcing respiratory dysregulation as a core early phenotype.

The mechanistic basis of hypotonia, motor developmental impairments, and respiratory morbidity in PURA syndrome is likely heterogeneous. PURA is expressed in many tissues, including the brain and peripheral nervous system [3]. Recent reports have challenged a purely central basis for symptoms by describing congenital myasthenic syndrome-like (CMS-like) physiology in patients with PURA syndrome [3].

Mroczek et al. [4] suggested CMS-like features in a cohort of 10 patients with PURA syndrome, where six had ptosis, and nine had myopathic facies. Other evidence comes from three case reports describing electrophysiological evidence of neuromuscular junction (NMJ) dysfunction (e.g., electro-decremental response on repetitive nerve stimulation (RNS)) with or without clinical response to agents that enhance neuromuscular transmission, including one patient with a positive response to salbutamol [4-7].

Here, we describe two patients with a positive response to NMJ-enhancing agents and summarize previously reported cases. Our findings provide key evidence to suggest that treatment of NMJ dysfunction, particularly with salbutamol, may provide benefit for patients with PURA syndrome.

## Case presentation

This is a case report of two patients with PURA syndrome treated with NMJ-directed therapy, with a review of three additional published cases. We included patients from two centres with genetically confirmed PURA syndrome who were identified as having used NMJ-enhancing agents such as pyridostigmine and salbutamol.

Clinical and genetic data

Clinical data for newly reported patients were obtained from electronic medical records. We extracted clinical data over time, including details of the neonatal phenotype and the evolution of tone, motor abilities, and respiratory phenotype. We documented treatment with NMJ-enhancing agents, including treatment used, dose reached, effect, and tolerability. Effect was determined relative to pre-treatment clinical features, both descriptively and quantitatively where available.

Genetic testing

Trio-whole exome sequencing (trioWES) is performed using Illumina sequencing by two independent laboratories. Sequencing read alignment (GRCh38), variant calling, annotation, and prioritization are done using in-house bioinformatics pipelines according to the phenotypes provided. Genetic variants were assessed for pathogenicity according to the American College of Medical Genetics and Genomics guideline [8]. Only pathogenic and likely pathogenic variants were included.

Ethics

Written informed consent was obtained from the parents of the two newly reported patients.

Case 1

Patient 1 was currently a 14-month-old child born to non-consanguineous healthy parents with a well-appearing first week. On day 9, he presented with episodes of cyanosis during feeding. Upon hospital admission, he was noted to have repeated transient apneas, which resolved with supplemental oxygen (1L O_2_/min). In the third week of life, he had increasing apneas requiring non-invasive positive pressure ventilation (NIPPV) and was stepped up to bilevel positive airway pressure (BiPAP) support in the fourth week due to type 2 respiratory failure. His physical examination showed peripheral hypotonia. No ptosis or myopathic facies was demonstrated. Sleep study at the fourth week of life showed a mixed pattern with a predominant central component. Nerve conduction studies showed a mixed motor polyneuropathy. RNS interpretation was limited due to interference in the neonatal intensive care (NICU) setting. trioWES identified a de novo 1.1kb heterozygous pathogenic deletion involving part of the PURA gene (c.333_*498del).

Pyridostigmine was initiated on D96 of life due to repeated desaturations despite BiPAP support. The dosage was gradually escalated to 5.8mg/kg/day over two weeks. There was a partial therapeutic response with fewer apneas, allowing weaning of respiratory support to high-flow nasal cannula (HFNC). Motor gains were not apparent. He had severe head lag and cannot turn. Salbutamol was added on D140 of life and titrated up to 0.3 mg/kg/day. Within two weeks, there was a resolution of apneas allowing four hours per day ventilation-free, though partially dependent on the laryngomalacia obstructive component. He was noted to have stronger anti-gravity movements, able to make a partial turn and more extension thrust, though it was not reflected in standardized measures. The Alberta Infant Motor Scale (AIMS) [9] was 5/58 (5th percentile) at baseline; 10/58 (5th percentile) at two weeks, with optimized dosage of salbutamol at five months of age. Pyridostigmine was ceased after one month of use, and the respiratory gain was maintained with no further apneas observed. At the follow-up visit at 10 months, his AIMS measure was 12/58 (5th percentile - 24/58). He had neuroregression after epileptic spasm onset at nine months.

Case 2

Patient 2 was a 17-month-old child at present, born to non-consanguineous healthy parents. He required immediate resuscitation for poor respiratory effort at birth. He was noted to have significant hypotonia with severe feeding issues requiring nasojejunal (NJ) feeding. At six weeks of age, he was transferred to a tertiary NICU with severe hypotonia and worsening apneas requiring NIPPV and then BiPAP. He was diagnosed with mixed sleep-disordered breathing. He had a prominent forehead and round cheeks, with a slight myopathic facies. Trio WES identified a de novo pathogenic frameshift variant in thePURA gene, c.148dup; p.(Ala50Glyfs*151).

He had a prolonged NICU admission, ultimately being discharged home at age five months. He had severe hypotonia, with frog-legged posture and minimal antigravity movements, ongoing BiPAP requirement, and was solely dependent on NJ feeding. At age 11 months, he had minimal improvement. He had incomplete head control and could not turn his head to visually track without considerable head and neck support.

Nerve conduction studies showed normal motor and sensory responses. Slow repetitive stimulation at 3Hz showed no electro-decremental response at rest; repeat stimulation after muscle activation was not tolerated. Salbutamol was commenced at 11 months and 15 days and stepped up to 0.24 mg/kg/day over three months. At the higher doses, he developed excessive startling, disturbed sleep, and tachycardia compared to baseline, which resolved after dose reduction to 0.19 mg/kg/day.

After 10 weeks of treatment (on 0.18 mg/kg/day), he had improved head control, was better able to tolerate a supported sitting position, and had more antigravity movements and improved endurance. The Children's Hospital of Philadelphia Infant Test of Neuromuscular Disorders (CHOP INTEND) [10] score improved from 50/65 (baseline) to 54/65 at day 70 of treatment. Timed anti-gravity movement of upper and lower limbs substantially improved. At baseline, he was able to hold his arms elevated (while supine and at 90 degrees shoulder flexion) for 3.5 minutes over the left arm and four minutes over the right arm. This improved to over 10 minutes (both arms) at day 70 of treatment.

By day 150 after salbutamol commencement, he was able to sit unsupported. Six months after commencement, he continues on salbutamol at 0.19 mg/kg/day. He remains dependent on NJ feeding, but is tolerating increasing amounts of oral intake, taking 240 grams of puree per day. He remains on BiPAP, although he tolerates daytime sleep and occasional nights without it, with no desaturations, work of breathing or snoring reported. Repeat polysomnography assessment is awaited to determine whether ongoing ventilatory support is required.

## Discussion

In addition to our two patients, we identified three literature-derived cases from two published reports [9,10]. A systematic search was carried out on PubMed and MEDLINE, using the keywords “PURA”, “PURA syndrome”, “pyridostigmine”, and “salbutamol” to extract data on previously published patients. Only those published in English were included. Last search date was 30th April, 2026.

Their clinical characteristics are summarized in Table 1, therapeutic response in Table 2, and treatment response in Table 3. Of the five patients, three were male and two female. All five had neonatal hypotonia, weakness, and poor feeding. All had apnea and/or respiratory failure, requiring respiratory support in young infancy. Three had electrophysiologic evidence of NMJ dysfunction, and two had limited studies. Pyridostigmine was tried in four patients, with clear benefit reported in one [9]. Salbutamol was associated with benefit in all three patients in whom it was trialled [10].

**Table 1:** Summary of genotype and pre-treatment phenotype for five infants with PURA syndrome treated with NMJ-directed therapy PURA: purine-rich element-binding protein A; BiPAP: bilevel positive airway pressure; CMAP: compound muscle action potential; CMS: congenital myasthenic syndrome; EMG: electromyography; GDD: global developmental delay; NCS: nerve conduction study; NICU: neonatal intensive care unit; NIPPV: noninvasive positive pressure ventilation; NG: naso-gastric; NJ: naso-jejunal; NMJ: neuromuscular junction; NS: not stated; PUR-I/-II/-III: PUR repeat domains I/II/III; RNS: repetitive nerve stimulation

Patient	Sex	Age at report (months)	PURA variant; zygosity inheritance; ACMG classification; PUR repeat domain affected	Neonatal phenotype	Post-neonatal/pre-treatment phenotype					Nerve conduction studies and electromyography (age performed)
					Age (months)	Motor phenotype	Respiratory phenotype	Feeding	Other	
P1	M	14 m	c.333_*498del; p.? Heterozygous; De novo pathogenic; PUR-I to PUR-III domains	Evolving neonatal hypotonia, respiratory failure requiring BiPAP	3	Severe hypotonia with poor head control, no rolling, limited antigravity movement	Mixed central and obstructive apnea, BIPAP dependence, discussion of tracheostomy	NG fed	Severe GDD	NCS: mixed motor polyneuropathy; RNS limited by ICU interference
P2	M	17 m	c.148dup; p.(Ala50Glyfs*151) Heterozygous; De novo pathogenic; PUR-II and PUR-III domain	Neonatal hypotonia, dysphagia requiring tube feed, respiratory failure needing ventilatory support from birth, poor thermoregulation (now resolved)	11	Severe hypotonia, very poor head control, not rolling, limited antigravity movements of limbs	Mixed sleep-disordered breathing, ongoing BiPAP dependence during sleep	NJ fed	Severe-profound GDD, dysconjugate eye movements, no epilepsy	RNS limited; no definite decrement; normal EMG of tibialis anterior (11 months)
P3, Wyrebek et al. [6]	F	10 m	c.697_699del; p.Phe233del Heterozygous; Pathogenic; PUR-III	Severe neonatal hypotonia, dysphagia, poor thermoregulation, apneic episodes	2	Significant head lag, intermittent tongue fasciculation	Severe apnea; mixed sleep apnea; NIPPV dependence; discussion of tracheostomy	NG fed	GDD, visual impairment	Slow (3Hz), fast (30Hz) RNS and needle EMG, suggestive of irritable myopathy. Possible mixed, pre- and synaptic NMJ
P4, Qashqari et al. [7]	M	NS	c.313_318del; p.Ser105_Val106del Heterozygous; De novo; Likely pathogenic; PUR-I	Respiratory failure needing ventilatory support from birth, hypotonia, fluctuating muscle weakness; bulbar weakness, high-arched palate, retrognathia, joint hyperlaxity	NS	Hypotonia, fluctuating muscle weakness	Episodic apnea; NIPPV dependence	NG fed	-	Double-peak CMAP and 49% decrement at 3 Hz; CMS-like and slow channel-related pattern
P5, Qashqari et al. [7]	F	60 m	c.616_618delATC; p.Ile206del Heterozygous; De novo; Likely pathogenic; (PUR-II)	Neonatal hypotonia; fluctuating weakness and excessive startle response, dysphagia requiring tube feeding	3	Hypotonia, fluctuating muscle weakness	Episodic apnea; respiratory support	NS	-	3Hz RNS showed 46% decrement response and rebound increment (50%) at 20Hz on D25, suggestive of CMS like pattern.

**Table 2:** Exposure to and response to pyridostigmine and salbutamol across the five cases HFNC: high-flow nasal cannula; NIPPV: noninvasive positive pressure ventilation; O_2_: oxygen; TDS: three times daily; AIMS: Alberta Infant Motor Scale; CHOP INTEND: Children's Hospital of Philadelphia Infant Test of Neuromuscular Disorders

Patient age at report	Pyridostigmine exposure age at commencement dose	Pyridostigmine response	Pyridostigmine adverse effects	Pyridostigmine use ongoing?	Salbutamol exposure age at commencement dose	Salbutamol response	Salbutamol adverse effects	Salbutamol use ongoing?
P1 14 months	Yes, at age three months, titrated to ~5.8 mg/kg/day	Partial: weaned to HFNC, persistent transient apnea	None reported	No	Yes; age: 4.5 months 0.3 mg/kg/day	Improved; No further apneas with four hours off HFNC/day -> off at 14 months; Stronger anti-gravity movements: Partial turn and more extension thrust. Formal assessments: AIMS 5/58 (5th percentile) at 3.5 months (baseline) -> 10/58 (5th percentile) at five months of life.	None reported	Yes (9.5 months of use at present)
P2 17 months	No	Not applicable	Not applicable	Not applicable	Yes; age 11.5 months, 0.19 mg/kg/day	Improved; Unsupported sitting by day 150 of treatment, remains NJ feed dependent but tolerating greater oral intake (~240g puree per day); Awaiting results of recent polysomnography to determine ongoing need for BiPAP Formal assessments: Timed antigravity movements: UL: Left 3mins 28 sec and Right 4 mins (Baseline) -> Both >10 mins (D70 of treatment); LL: 45s (Baseline) -> 1 min 45 secs (D70); CHOP INTEND: 50/65 (Baseline) → 54/65 (D70)	Tachycardia, increased startle, sleep disturbance. Resolved with dose reduction (from 0.24 mg/kg/day to 0.19 mg/kg/day)	Yes (six months of use at present)
P3, Wyrebek et al. [6]	Yes, every six hours from day 64 (dose not specified)	Improved tone and apnea within 24 h; weaned off NIPPV	None reported	On pyridostigmine at 10 months	No	Not applicable	Not applicable	-
P4, Qashqari et al. [7]	Yes	Worsening: respiratory deterioration with profound apnea and bradycardia	Profound apnea and bradycardia	No	Yes	Improved: apnea resolved; NIPPV discontinued; strength improved	None reported	Not reported
P5, Qashqari et al. [7]	Yes	No response; stopped after several days	None reported	No	No	Not applicable	Not applicable	Not applicable

**Table 3:** Treatment-response summary in this cohort: two original cases and three literature derived cases [6,7]

Treatment	Exposed cases	Improved	Partial	No response	Worsening	Adverse effects reported
Pyridostigmine	4/5	1/4	1/4	1/4	1/4	1/4
Salbutamol	3/5	3/3	0/3	0/3	0/3	1/3 (resolved with dose reduction)
Sequential or combined pyridostigmine and salbutamol	2/5	2/2 improved after salbutamol	N/A	N/A	N/A	0/2 salbutamol adverse effects reported in sequential cases

This case series expands the emerging evidence that NMJ dysfunction may contribute to respiratory and motor morbidity in people with PURAsyndrome and suggests that treatment with salbutamol may provide clinically meaningful benefit for some patients.

Although dysfunction of central mechanisms for control of tone, movement, and respiration is an important mechanism inPURA syndrome, the clinical pattern and treatment responses observed in our case series add to previous literature suggesting that NMJ involvement can also contribute to respiratory failure as well as delayed motor acquisition.

Our two newly reported patients had objective assessments prior to and during treatment with NMJ-enhancing agents. These objective measures strengthen the clinical impression of therapeutic effect and help address limitations of previous reports, in which response was described subjectively without standardized respiratory or motor functional measures.

In Case 1, pyridostigmine was associated with a partial reduction in apneic episodes, but clinically meaningful improvement occurred after the addition of salbutamol, with apneic events and stronger antigravity movements. In Case 2, salbutamol use led to measurable motor gains, including improved head control, unsupported sitting, increased timed antigravity movement tasks, and improvement in CHOP INTEND score.

The response pattern to pyridostigmine was conflicting. Among the four exposed patients, two had improvement, one showed no response, and one worsened with profound apnea and bradycardia. This variability suggests that PURA-associated NMJ dysfunction may not represent a single uniform synaptic defect and that acetylcholinesterase inhibition may be beneficial in some patients but ineffective or potentially harmful in others. Previous electrodiagnostic studies [9] are suggestive of both pre-synaptic and post-synaptic involvement. The two cases with non-response or worsening with pyridostigmine both had electro-decremental response at low frequency RNS. This pattern is described in COLQ-related CMS and slow-channel-related CMS, conditions where pyridostigmine is also typically not beneficial. In contrast, one patient with PURAsyndrome had a similar electro-decremental response at low-frequency RNS but had reported benefit with pyridostigmine. We advise caution with pyridostigmine inPURA syndrome, with use considered at the individual level rather than empirically trialed in all patients. If trialed, we suggest cautious initiation and close cardiorespiratory monitoring.

In contrast, all three salbutamol-exposed patients in this cohort improved. Salbutamol has established therapeutic use in selected CMS, particularly those involving impaired postsynaptic stability or NMJ maintenance, e.g., SLC5A7-, COLQ-, CHRNE-, DOK7-, MUSK-, COL13A1-, and GMPPB-related CMS [11]. The apparent benefits observed in patients with PURA syndrome may therefore indicate that PURAdysfunction affects synaptic development, maintenance, or compensatory neuromuscular signaling rather than acetylcholine availability alone.

Electrodiagnostic findings in this cohort were variable. In the two newly reported cases, electro-decrements were not demonstrated with RNS. However, both studies were limited due to young age (making RNS technically challenging) and an intensive care environment where study findings can be contaminated by equipment artefact. These findings highlight that a normal or inconclusive RNS study should not exclude NMJ involvement in PURA syndrome. Judicious consideration of salbutamol use can be contemplated in those with severe respiratory or motor symptoms. It is known that with time, patients with PURA syndrome will outgrow their respiratory insufficiency, and non-invasive ventilation is rarely required beyond infancy [12]. However, prompt consideration of salbutamol use could lead to earlier respiratory stabilization and acceleration of gains in motor and feeding abilities. Respiratory stabilization is particularly important, as it may potentially facilitate earlier hospital discharge in admitted infants, with favorable outcomes on health, family bonding, and neurodevelopmental stimulation.

Our findings also support the inclusion of CMS-like phenotype work-up for patients with PURA syndrome, including detailed neuromuscular examination, serial respiratory and feeding assessment, and nerve conduction studies with RNS where feasible. If NMJ-directed therapy is trialed in patients with PURA syndrome, objective comparison with pre-treatment baseline is critical. Treatment response should ideally be monitored using predefined respiratory endpoints such as ventilatory support requirements, apnea burden, or polysomnography parameters, together with standardized motor scales such as CHOP INTEND or AIMS.

Limitations

Several limitations should be acknowledged. First, the cohort is small, with heterogeneity in clinical detail documentation, electrophysiologic methods requisition, treatment timing, and outcome reporting. Second, limited use of objective outcome measures makes it difficult to compare the effects of treatment in some individuals. Third, maturation of respiratory control may confound apparent respiratory benefits of treatment, particularly when therapy is introduced during late infancy/early childhood when the need for non-invasive ventilation may be outgrown. Fourth, salbutamol was introduced after pyridostigmine in two cases, making it difficult to separately delineate their individual treatment effects, although in one of these patients, pyridostigmine was able to be ceased without worsening. Finally, we are not able to determine the proportion of patients with PURA syndrome who may derive benefit from salbutamol. We are not aware of additional patients in whom salbutamol has been trialed, but publication bias may have enriched the literature for treatment-responsive cases.

## Conclusions

Despite these limitations, this series provides further evidence that NMJ-directed therapy may lead to a clinically relevant benefit in patients with PURA syndrome. The improvement observed after salbutamol exposure in three cases supports the hypothesis that PURA syndrome may include a treatable CMS-like component in some individuals. These findings justify systematic multi-centre prospective studies of salbutamol in people with PURA syndrome, using standardized electrophysiology measures, polysomnography, respiratory outcome measures, and serial motor assessments. Until such data are available, treatment decisions should be individualized and interpreted within the broader context of PURA syndrome as a multisystem neurodevelopmental disorder involving both central and peripheral mechanisms.
